# Statistics of Affections of the Upper Jaw

**Published:** 1858-01

**Authors:** 


*Statistics of Affections of the Upper Jaw.*—Mr. Oscar Heyfelder, of Munich, has published a statistical account of 456 cases of the superior maxilla. Of these, there were 41 of necrosis; 22 of caries; 1 of sclerosis; 6 of fibrous tumor; 48 of sarcoma; 7 of enchondroma; 13 of exostosis; 7 of osseous growth; 3 of cancroid; 74 of carcinoma; 41 of indeterminate tumors; 34 of accumulation of liquids; 8 of teeth in the antrum; 125 of indeterminate affections.

Of 158 cases in which the sex of patients was known, 85 occurred among males and 73 among females.

Of 136 in which the age is stated, 9 were from 4 to 10 years; 31 from 11 to 20; 30 from 21 to 30; 19 from 31 to 40; 20 from 41 to 50; 16 from 51 to 60; 8 from 61 to 70; and 3 from 71 to 76. Of 197 cases, 92 concerned the right bone, 96 the left, and 9 both. This surgeon has made 368 resections complete and partial of this bone; in 128 the result is not mentioned; in 66 the patient died or the disease returned, and in 116 the result was favorable. In 9 cases of complete resection of both bones, 4 were entirely successful.

